# Validation of instruments about family presence on invasive procedures and cardiopulmonary resuscitation in pediatrics

**DOI:** 10.1590/1518-8345.2368.3046

**Published:** 2018-11-14

**Authors:** Cristiana Araújo Guiller Ferreira, Flávia Simphronio Balbino, Maria Magda Ferreira Gomes Balieiro, Myriam Aparecida Mandetta

**Affiliations:** 1 Hospital São Paulo, Unidade de Terapia Intensiva Neonatal, São Paulo, SP, Brazil.; 2 Universidade Federal de São Paulo, Escola Paulista de Enfermagem, São Paulo, SP, Brazil.

**Keywords:** Family, Patient Care Team, Pediatrics, Invasive Procedures, Cardiopulmonary Resuscitation, Nursing

## Abstract

**Objective:**

to develop and validate instruments to identify health professionals’ beliefs related to the presence of the child’s family in invasive procedures and in cardiopulmonary resuscitation.

**Method:**

study based on Psychometrics to conduct the theoretical, empirical and analytical stages, developed in a neonatal unit of a university hospital. The two instruments were constructed based on the literature and applied to 96 health professionals.

**Results:**

the Cronbach’s Alpha of the instrument related to the professionals’ beliefson invasive procedures was 0.863 and the instrument on cardiopulmonary resuscitation was 0.882. In both instruments, the tests performed indicated a correlation between the items. From the factorial analysis, four factors were generated: (1) benefits of the presence of the family; (2) impairment for professional practice; (3) strategies for the inclusion of the family; and (4) limitation of learning and decision making by the professional.

**Conclusion:**

the instruments analyzed obtained a good internal consistency and are indicators of the professionals’ beliefs with the potential to evaluate the quality of family care in this context.

## Introduction

The family presence during invasive procedures (IP) and cardiopulmonary resuscitation (CPR) has been investigated in the last 20 years and recommended in emergency care settings by international organizations, such as the American Heart Association (AHA) and the American Association of Critical Care Nurses (AACN)^1^ ^-^ ^2^.

The conceptual definition of the family presence during IP and CPR is the presence of one or more family members in a place with physical and/or visual contact with the patient. However, the decision of the health professionals to provide the family presence is based on their beliefs, values and knowledge^3^ ^-^ ^4^.

In the pediatric context, it is relevant to understand the professionals’ beliefs about the family in order to identify those that may be restrictive to their participation in the health care of their children so that actions can be proposed to help them transform them. Beliefs are the lenses we use to see the world and guide our choices, behaviors and feelings^5^.

In a cross-sectional study conducted in Brazil with 46 participants^6^regarding health professionals’ opinions regarding the family presence in the pediatric emergency room, the authors concluded that the medical team was more favorable than the nursing professionals. In addition, there was a statistically significant association between having less than ten years of time of graduation and the acceptance of this practice. These results point to the need to raise professionals’ awareness in this area, especially those with more time of graduation, to facilitate the presence of a family member next to the child during care in these circumstances.

In a review of the literature on this subject^7^, the authors identified restrictive beliefs of professionals, such as that the family hinders the team in performing the procedures; the family does not understand what is being done; the family suffers from witnessing the procedures performed on the child; the team feels pressured for decision making by the presence of the family; the team loses concentration during the performance of the procedures; and the professionals under training may have their learning damaged. Thus, they proposed four fundamental actions to promote the presence of the family: a) offering programs to raise awareness on the family’s participation in care; b) qualifying the team to include the family in those circumstances; c) developing a declared institutional policy; and d) providing institutional support for the family during interventions.

There is little or no study that supports the restrictive beliefs of professionals indicating that the presence of a family member is detrimental to the patient, the family or the health team. On the contrary, there are studies that validate this recommendation and demonstrate its benefits, which are: decrease in family anxiety; little or no family interference in health care; facilitation of the family’s grieving process when the outcome is negative; and improvement in the technical ability of health professionals^2^ ^,^ ^8^ ^-^ ^11^. 

For the American Association of Critical Care Nurses, there is strong scientific evidence that the family prefers to be present during the IP and CPR^8^.In Brazil, in most institutions, there is a restriction on the permanence of the family during the IP and CPR. However, there are reports of families’ discontent when they are invited to leave the treatment room during an IP or CPR due to lack of information and the removal of the child, often over long periods. To transform this reality, it is necessary to promote a change in the model of care, in which the family is included as a collaborator and participates in the decision-making regarding their child. The Patient and Family-Centered Care Model advocates that health professionals work in partnership with the family, offering information and negotiating care^12^ ^-^ ^13^ ^).^

Therefore, it is necessary to listen to families and professionals about their perspectives regarding the permanence of the family next to the child, experiencing critical situations or undergoing invasive procedures. The results will enable proposing interventions that modify this reality. In the literature, there are several studies addressing this issue using several methodological approaches whose results can contribute to the theoretical basis of instruments to measure family participation in invasive procedures and in cardiopulmonary resuscitation^3^ ^-^ ^4^ ^,^ ^6^ ^-^ ^11^.

Instruments to measure psychosocial phenomena have been developed in the literature by scholars and are objective tools for nurses to know and support their interventions^14^ ^-^ ^16^.In Brazil, there are no studies addressing the perspective of health professionals on the subject with the use of objective instruments. For this reason, it is necessary to develop and validate instruments that enable evaluating the professionals’ beliefs and perceptions about this issue. From these indicators, it will be possible to propose educational and management interventions to improve care with the family in critical situations.

The research objectives are to develop and validate instruments to identify health professionals’ beliefs related to the presence of the child’s family in invasive procedures and in cardiopulmonary resuscitation.

## Method

In this research, the procedures of a methodological research based on Psychometry, according to Pasquali, were adopted to conduct the theoretical, empirical and analytical steps^17^ ^-^ ^18^.

At the theoretical stage, an integrative review was carried out^7^ ^)^ with the aim of identifying indicators of professionals’ beliefs and perceptions about the family presence during the IP and CPR. Subsequently, the theoretical material produced guided the constitutive and operational definitions of the instruments. From then on, the items of the instruments were elaborated based on the criteria of breadth, behavioral balance, simplicity, clarity, relevance, accuracy, modality, typicity, objectivity, variety and credibility^17^ ^-^ ^18^.

The content of the instruments was validated by a Judging Committee, composed of experts on the subject, namely two nurses, a physician, a physiotherapist and a psychologist with more than three years of experience and working in neonatal and pediatric units. These experts evaluated the tools for semantics, comprehension of questions, writing and conceptual relevance. The members filled out a questionnaire with spaces to answer each item in relation to their relevance and the issuance of suggestions. The Delphi technique was adopted to reach agreement between the judges^19^.

The empirical step was carried out in a public service of the city of São Paulo, linked to a higher education institution. The inclusion criteria of the participants were: being health professionals and having worked as teachers and/or care providers for at least one year in the neonatal unit. Twenty professionals participated in the pilot test through test-retest and the 96 participated in the clinical application of the instruments.

In the analytical stage, we verified the reliability, validity and estimation of item parameters related to the identification of the health professionals’ beliefs and perceptions about the family presence in emergency situations and invasive procedures. 

The sample calculation was set according to the criterion ‘item/subject ratio’, as recommended in the literature, and we adopted a minimum proportion of five participants for each item of the instrument^17^ ^-^ ^18^.To collect the data, an in-person invitation was made to the research participants, who were informed about the purpose of the study and the voluntary nature of their participation. After acceptance, manifested through the signing of the Informed Consent Form, the instruments were delivered respecting the ethical principles that rule research involving human beings, as defined by Resolution of the National Health Council 466/2012^20^. The research project was approved by the Ethics and Research Committee of the institution under number 332543.

The psychometric tests used for data analysis were the Kappa Concordance Index, the Intraclass Correlation Index (ICC), the Cronbach’s Alpha, the Test for Equality of Two Proportions, the Kaiser-Meyer-Olkin (KMO), the Bartlett’s test and the Factorial Analysis. To compare the qualitative variables, the Analysis of Variance (ANOVA) test was used. The significance level adopted for the tests was 5% (α = 0.05) and the statistical package used was SPSS for Windows, version 17.0 (SPSS Inc. Chicago, Illinois), Minitab 16 and Excel Office 2010.

## Results

The literature review resulted in the proposition of version I of the instruments that were named “Health Professionals’ Beliefs on the Family presence during Invasive Procedures - HPBF/IP and Beliefs of Health Professionals’ Beliefs on the Family presence in Cardiopulmonary Resuscitation - HPBF/CPR”.

After the content was validated, there were changes in the language and format of version I of each of the instruments that, once incorporated into the text, were re-assessed. Thus, there was 100% of agreement between the judges, in two rounds, which resulted in the version II of each of the instruments.

Each of the approved instruments has 18 questions - seven referring to the restrictive beliefs about the construct analyzed (questions 3 to 9). In order to analyze the final result, they must be interpreted in an inverted way. The answers to the questions are distributed on a Likert scale containing the options ‘I strongly disagree’, ‘I disagree’, ‘Neutral’, ‘I agree’ and ‘I strongly agree’.

In the test-retest of the instruments, performed in the empirical stage, we found that the intraclass correlation index (ICC) for the IP and CPR was 53.3%, p-value <0.001. The Cronbach’s alpha value for the HPBF/IP instrument was 0.774, both in the test and in the retest, and for the HPBF/CPR instrument, it was 0.821 in the test and 0.762 in the retest, indicating a median and good internal consistency.

In the clinical application of version II of the instruments, of the 96 participants, most were female (92, 95.8%), single (50; 52.0%) and without children (55; 57.3%). The highest frequency of age was between 31 and 45 years (45, 46.9%). As for professional training, 15 (15.6%) participants were nurses, 20 (20.8%), nursing technicians; 11 (11.5%), nursing assistants; 21 (21.9%), physicians; seven (7.3%), physiotherapists; one (1.0%), psychologist; and 21, postgraduate students - 18 (18.8%) resident physicians and three, 1%) resident physiotherapists. Among the participants, 74 (77.1%) had worked three or more years in neonatology, and 51 (53.1%) had specialization in the pediatric area. The majority reported having experience with the hospitalization of a member of their family and knowledge about the theme acquired during their graduation.

In this step, the Cronbach’s Alpha of the HPBF/IP instrument was 0.883, and of the HPBF/CPR instrument was 0.882, which demonstrates a good internal consistency.

The result of KMO for the HPBF/IP instrument was 0.849, and for HPBF/CPR, 0.843, which made it possible to perform the factorial analysis. The Bartlett’s test was significant, that is, the null hypothesis was rejected and, for this reason, we can state that the correlation matrix is different from the identity matrix. Therefore, there is a correlation between the data in the two instruments.

The 18 questions of each instrument generated four factors (groups of questions or domains) in each, in which the total variability was explained for HPBF/IP in 63.8%, and for HPBF/CPR in 62.4%. Statistically, it was considered a median to good value (Tables 1 and 2).

**Table 1:** Factorial load of the questions in each IP factor* - São Paulo, SP, Brazil - 2015^†^

	Questions (Q)	Factor 1	Factor 2	Factor 3	Factor 4
Benefits of the presence of the family	Q.14 - The family presence can help in the grieving process if the child does not survive.	0.770			
	Q.15 - The family presence during the IP* without success favors their participation in the moments experienced by the child.	0.768			
	Q.13 - The family presence can strengthen the bond between the family and the health team.	0.750			
	Q.11 - The family presence can help them understand what is being done with the child.	0.748			
	Q.9 - The family’s emotional distress can be reduced when they are present in the IP*.	0.721			
	Q.12 - The family presence may be beneficial to the child during IP*.	0.703			
	Q.1 - The multidisciplinary team should assure the family the opportunity to decide whether or not to be present during the IP*.	0.674			
	Q.3 - The family presence is beneficial for the family member to understand the decision-making of the team.	0.621			
	Q.10 - The family presence can help them accept the decision-making of the team.	0.580			
Impairments for professional practice	Q.6 - The family presence can contribute to the team losing concentration.		0.869		
	Q.7 - The family presence can generate insecurity and anxiety in the team and affect their attitudes.		0.773		
	Q.5 - The family presence can influence the prolongation of the procedure by the team.		0.760		
	Q.4 - The family presence can interfere in the decision-making adopted by the team.		0.713		
Strategies to include the family	Q.18 - The health professionals should be trained to include the family.			0.862	
	Q.17 - The neonatal unit must have a written protocol on the presence of the family.			0.803	
	Q.16 - A member of the health team should be chosen to meet the family’s needs.			0.787	
	Q.2 - The multidisciplinary team should make the decision to invite the family to witness the IP*.			0.461	
Limitation of learning and decision-making	Q.8 - The family presence can impair the learning of professionals.				0.927

*IP - Invasive procedure; †Extraction method: principal component analysis. Rotation method: Varimax with Kaiser Normalization. Rotation converged in 5 interactions.

**Table 2:** Factorial load of the questions in each CPR factor*. São Paulo, SP, Brazil, 2015^†^

	Questions (Q)	Factor 1	Factor 2	Factor 3	Factor 4
Benefits of the presence of the family	Q.15 - The family presence during the CPR* with no success favors their participation in the moments experienced by the child.	0.858			
	Q.13 - The family presence can strengthen the bond between the family and the health team.	0.811			
	Q.14 - The family presence can help in the grieving process if the child does not survive.	0.794			
	Q.9 - The family’s emotional distress can be reduced when they are present in the CPR*.	0.753			
	Q.12 - The family presence may be beneficial to the child during CPR*.	0.689			
	Q.11 - The family presence can help them understand what is being done with the child.	0.647			
	Q.1 - The multidisciplinary team should assure the family the opportunity to decide whether or not to be present during the CPR*.	0.607			
Strategies to include the family	Q.17 - The neonatal unit must have a written protocol on the presence of the family.		0.841		
	Q.18 - The health professionals should be trained to include the family.		0.728		
	Q.16 - A member of the health team should be chosen to meet the family’s needs.		0.594		
	Q.2 - The multidisciplinary team should make the decision to invite the family to witness the CPR*.		0.557		
	Q.10 - The family presence can help them accept the decision-making of the team.		0.391		
Limitation of learning and decision-making	Q.5 - The family presence can influence the prolongation of the procedure by the team.			0.840	
	Q.4 - The family presence can interfere in the decision-making adopted by the team.			0.691	
	Q.8 - The family presence can impair the learning of professionals.			0.566	
Impairments for professional practice	Q.7 - The family presence can generate insecurity and anxiety in the team and affect their attitudes.				0.838
	Q.6 - The family presence can contribute to the team losing concentration.				0.573
	Q.3 - The family presence is beneficial for the family member to understand the decision-making of the team.				0.528

*CPR - Cardiopulmonary resuscitation; †Extraction method: Principal component analysis. Rotation method: Varimax with Kaiser Normalization. Rotationconverged in 7 interactions.

In the HPBF/IP instrument, factor 1 represents the professionals’ beliefs related to the benefits of the presence of the child’s family during the IP; factor 2 describes the restrictive beliefs related to the losses to the practice of the health professional; factor 3 refers to the beliefs about strategies to promote family inclusion; and factor 4 exposes the professional’s restrictive beliefs regarding the limitation of self-learning and decision-making in the presence of the child’s family during the IP.

In the HPBF/CPR instrument, factor 1 represents the professionals’ beliefs related to the benefits of the presence of the child’s family during the CPR; factor 2 concerns beliefs about strategies to include the family in CPR; factor 3 exposes the professional’s restrictive beliefs regarding the limitation of learning and decision-making in the presence of the child’s family during CPR; and factor 4 describes the restrictive beliefs related to the losses to the practice of the health professional.

In the HPBF/IP instrument, factor 1 presented 33.2% of the total variability and is composed of nine questions (Q): 1, 3, 9, 10, 11, 12, 13, 14 and 15. The question with the highest factorial load (0.770) was 14 (The family presence can help in the grieving process if the child does not survive after IP). However, in factor 4, Q.8 (The family presence can impair the learning of professionals during the IP) was the one with the highest factor load (0.927), despite the variability of 6%.

In the HPBF/CPR instrument, the factor 1 result alone held 36.5% of the data variability and consists of questions (Q) 1, 9, 11, 12, 13, 14 and 15. Among the seven items, the highest factor load (0.858) was at Q.15 (The family presence during the CPR with no success favors their participation in the moments experienced by the child), which indicates its higher correlation with the evaluated construct.

In the HPBF/CPR instrument, Q.10 (Q.10 - The family presence can help them accept the decision-making of the team during CPR) had a low factorial load (0.391). Therefore, a reanalysis was necessary to remove this item or a new arrangement among the existing factors.

With the created factors, it was possible to calculate their coefficients for each research participant. In the HPBF/IP instrument, a combination of factor 1 with titration (p-value 0.005) was found. There was a positive association between factor 1 and professionals with a PhD degree (mean of 0.573) and negative association for those with a middle level of education (mean of -0.677).

In the HPBF/CPR instrument, there was an association of factor 1 with titration (p-value 0.002), with a positive result for professionals with a PhD degree (mean of 0.787) and negative for those with a middle level of education (mean of 0.787). Regarding the ‘profession’ category (p-value 0.003), there was highlight for physicians (mean 0.556), nursing assistants (mean of -0.560) and professionals with previous experience of hospitalization of a relative (p-value 0.038). These latter responded positively (mean of 0.098).

Regarding the distribution of the answers to the items related to the beliefs about strategies to effect the family presence in the unit, there was agreement among the respondents regarding the need for written protocols to guide the practice (88.6% inHPBF/IP, 82.1% inHPBF/CPR); training of the team (89.6% inHPBF/IP, 76.8% inHPBF/CPR); choice of a member to accompany the families (80.2% inHPBF/IP, 86.3% inHPBF/CPR) and invitation for the family to attend the event (53.1% inHPBF/IP, 51.6% inHPBF/CPR).

The HPBF/IP instrument had a significant association with the demographic variables in the following questions: Q.12 (The family presence may be beneficial to the child during the IP) for professionals aged 31 to 55 years (p -value 0.023); Q.4 (Q.4 - The family presence can interfere in the decision-making adopted by the team during the IP) was associated with knowledge about the theme (p-value 0.004) and Q.10 (The family presence can help them accept the decision-making of the team during the IP) with the professional category (p-value 0.014) for physicians, nurses and nursing assistants.

Five questions had associations with professional qualifications: Q.10 (The family presence can help them accept the decision-making of the team during the IP), with professionals who have PhD, Master’s degree and newly graduates (p-value <0.001); Q.11 (The family presence can help them understand what is being done with the child during the IP), with those with higher education (p-value 0.005); Q.12 (The family presence may be beneficial to the child during the IP), with those with higher education (p-value 0.047); Q.13 (The family presence can strengthen the bond between the family and the health teamduring the IP),with PhD, masters and specialists (p-value 0.042); and Q.14 (The family presence can help in the grieving process if the child does not surviveafter the IP) with those with higher education (p-value 0.032).

In the HPBF/CPR instrument, there was a significant association related to previous experience with the hospitalization of a family member in the questions: Q.1 (The multidisciplinary team should assure the family the opportunity to decide whether or not to be presentduring CPR), between participants who reported previous experience of hospitalization in the family (p-value 0.016); and Q.5 (The family presence can influence the prolongation of the procedure by the teamduring CPR), with professionals who had not experienced this (p-value 0.029).

There was a significant association between the participants who are familiar with the theme and Q.13 (The family presence can strengthen the bond between the family and the health teamduring CPR) (p-value 0.049).

The demographic variable ‘occupation’ was associated with four questions: Q.1 (The multidisciplinary team should assure the family the opportunity to decide whether or not to be present during CPR), with nurses, physicians and nursing technicians (p-value 0.018); Q.12 (The family presence may be beneficial to the child during CPR), positive association with physicians and negative with nursing technicians and assistants (p-value 0.025); Q.13 (The family presence can strengthen the bond between the family and the health teamduring CPR),positive association with physicians and nurses and negative with nursing assistants (p-value <0.001); and Q.18 (The family presence can help them understand what is being done with the child during CPR), positive association with all occupations (p-value 0.011).

Four questions had associations with the titration variable: Q.11 (The family presence can help them understand what is being done with the child during CRP), positive association between physicians and masters and negative with the newly graduate (p-value 0.004); Q.13 (The family presence can strengthen the bond between the family and the health teamduring CPR), with physicians and masters (p-value 0.019); Q.14 (Q.14 - The family presence can help in the grieving process if the child does not survive after CPR), with higher-level professionals (p-value 0.006); and Q.18 (The health professionals should be trained to include the family during CPR), with postgraduate professionals (p-value <0.001).

## Discussion

The results obtained in the validation of the instruments on thehealth professionals’ beliefs of related to the family presence during IP and in CPR in the neonatal unit indicated satisfactory psychometric properties and therefore can be used in clinical practice.

The instruments obtained Cronbach’s alpha values indicating good internal consistency. This facilitated the understanding of the participants and the suitability of the content to the target population. Therefore, the items of both instruments refer to the construct being measured to meet the recommended in validation studies^17^ ^-^ ^18^.

In both instruments, the following domains representing the professionals’ beliefs about the family presence during IP and CPR were validated: I - Benefits of the presence of the family; II - Strategies for including the family; III - Limitation of learning and decision-making; and IV - Impairment of professional practice.

Domain I involves beliefs related to strengthening the bond between family and health team and child and emotional support for the family and child during the IP and CPR, favored by the presence and involvement of the family. In the clinical application, both instruments helped to identifying the professionals’ beliefs about the construct investigated. They believe that the presence of a family member in these two events is extremely important to strengthen the bonds among the family members themselves and between the professionals and the family so that they can witness the moments lived by the child.

It is important to highlight that the professionals’ facilitating beliefs identified with the application of the instruments corroborate the families’ beliefs evidenced in studies with families of children^21^ ^-^ ^24^ ^)^ and adult patients^25^ ^-^ ^27^. The authors concluded that when the family has the opportunity to stay with their loved one during emergency care their members often choose to continue and realize that their presence helps the mourning process in cases of death, as it provides a communication with the multiprofessional team and allows them to verify that all necessary efforts were made to save the patient’s life^6^ ^,^ ^25^ ^-^ ^27^.

Authors of a study that investigated the effect of parental presence and child’s distraction when using a toy during a painful procedure revealed that children who had someone close to them showed improvement in respiratory pattern, mean arterial pressure and heart rate, compared to children whose parents were absent. The children also reported less pain and felt less distressed. The authors concluded that the presence of parents is important to relieve children’s pain, stress and their negative behavior^21^.

In domain II, the objective is to identify the professionals’ beliefs related to the importance of institutional protocols and the qualification of the health team to keep the family next to their loved one during the IP and the CPR. In this study, most professionals believe that health facilities should have written protocols and a training program to implement this practice.

Studies^28^ ^-^ ^30^ ^)^ have pointed out that it is necessary to establish clear and written rules, such as designating a member of the health team to stay exclusively with the family, supporting their psychosocial needs with the aim of avoiding negative memories in the future and to previously evaluate the families’ emotional responses during the procedures in order to predict future interruptions in care.

It should be emphasized, however, that the American Association of Critical Care Nurses - AACN^31^warns that despite the recommendation of organizations, consensus conferences, policies and guidelines of a clinical practice to promote family inclusion in IP and CPR, only 5% of US, 8% of Canadians and 7% European critical care units have written policies. In the present research, the professionals with more time of schooling and previous knowledge on the subject are more open to the inclusion of the family during the IP and the CPR.

In France^32^ ^-^ ^33^, there is evidence that physicians with an average of 38 years of age with prior training related to the family presence during CPR are more favorable to this practice than nurses. 

Researches^12^ ^-^ ^13^ ^)^ have reinforced that the health team should encourage family participation in these processes, since they are important resources in the care that allow positive results in the patient’s clinical evolution. In this sense, the authors have pointed to the Patient- and Family-Centered Care as a potential model for conducting the practice of professionals, since it is based on the provision of information, respect for family dignity, participation in care and collaboration^12^. This model of care can meet the needs of patients and their families for receiving information and emotional support and being close to the team during a health-related crisis. From this perspective, health professionals should work towards recognizing that the family has the right to an explanation in a complete and appropriate way to understand the diagnosis and care given to their child.

Domain III refers to the restrictive beliefs of the health team regarding the family presence during the IP and CPR in relation to professionals’ learning and their capacity to make decisions.

In a survey conducted in the United States with 154 nurses on the family presence during CPR, the authors revealed that the self-confidence of these professionals was significantly higher among those who had completed advanced life support training, those who had participated in ten or more resuscitation events and those that were certified by specialist societies or nurses’ organizations^34^. The said study revealed that both high-level and mid-level health professionals believe that the presence of the child’s family during an IP and CPR does not interfere with learning, team focus, decision-making nor in the time of performance of procedures.

Domain IV is related to the beliefs about the damages to the professional practice when the family is present, such as anxiety, insecurity and loss of concentration during the IP and CPR.

These beliefs have been pointed out as those that motivate the professionals not to include the family in the IP and CPR. Intervention studies conducted in France revealed that, even after being aware that the family presence is extremely important during CPR, the medical staff maintains restrictive beliefs, such as that the presence of a relative can cause psychological damage, the procedure time becomes longer, the team loses concentration, there is risk of interference in medical management and that it increases the stress of the care team^32^ ^-^ ^33^.

Nurses who participated in that study^34^ ^)^ identified barriers to the family presence, such as the fear that it would interfere with procedures, lack of physical space and support for their members, fear of trauma, and increased anxiety.

As limitations of this study, we highlight the sample size, in which the item/participants ratio was considered at the minimum recommended; the inclusion of participants limited to a Brazilian city; the empirical stage of validation restricted to the neonatal context; the scarcity of national literature on the beliefs of health professionals regarding the family presence in critical situations; and the lack of care protocols in pediatric care services and of national consensus on the subject by expert societies, which made it difficult to construct the instruments.

## Conclusion

This study allowed the construction and validation of two instruments to identify the Health Professionals’ beliefs related to the Family Presence during Invasive Procedures - HPBF/IP - and the Beliefs of Health Professionals related to the Family Presence during Cardiopulmonary Resuscitation - HPBF/CPR, both with good internal consistency whose items refer to the same construct. The results of the factorial analysis indicated four factors with a load greater than 0.500, which were constituted in four domains of professionals’ beliefs. 

The proposed and tested instruments enable identifying the professionals’ beliefs about the events studied, to point out aspects that should be considered by the health team by taking care of the family as a partner in the health care of its members, and to contribute to improve the quality of care in this context. For this reason, they should be used in other national contexts, considering the country’s cultural diversity.

## References

[1] MacAlvin SS, Carew-Lyons A (2014). Family presence during resuscitation and invasive procedures in
pediatric critical care a systematic review. Am J Crit Care.

[2] American Association of Critical Care Nurses (2016). Practice Alert Family presence during resuscitation and invasive
procedures. Crit Care Nurse.

[3] Cathie G (2017). Family presence during resuscitation and invasive
procedures. Crit Care Nurse.

[4] Pasek TA, Licata J (2016). Parent advocacy group for events of resuscitation. Crit Care Nurse.

[5] Marshall A, Bell JM, Moules NJ (2010). Beliefs, suffering, and healing a clinical practice model for
families experiencing mental illness. Perspect Psychiatr Care.

[6] Mekitarian FFP, Angelo M (2015). Family's presence in the pediatric emergency room: opinion of
health's professionals. Rev Paul Pediatr.

[7] Ferreira CAG, Balbino FS, Balieiro MMFG, Mandetta MA (2014). Family presence during cardiopulmonary resuscitation and invasive
procedures in children. Rev Paul Pediatr.

[8] Kathleen LM, Jeff C, Susan E (2013). Family-centered care in the pediatric intensive care uniteline
family presenceduring invasive procedures and resuscitation. Pediatr Clin North Am.

[9] Nishisaki A, Diekena DS (2011). Mind the gap and narrowing it family presence during pediatric
resuscitation and invasive procedures. Resuscitation.

[10] Lederman Z (2016). Family presence during cardiopulmonary resuscitation
evidence-based guidelines? letter to editor. Resuscitation.

[11] Bossaert L, Perkins GD, Askitopoulou H, Raffay VL, Greif R, Haywood KL (2016). Reply to letter family presence during cardiopulmonary
resuscitation: evidence-based guidelines?. Resuscitation.

[12] Cruz AC, Angelo M (2011). Family centered care in pediatrics redefining
relationships. Cienc Cuidado Saúde.

[13] Dennis C, Baxter P, Ploeq J, Blatz S (2017). Models of partneship within family-centred care in the acute
paediatric setting a discussion paper. J Adv Nurs.

[14] Santos NC, Fugulin FMT (2013). Creation and validation of an instrument to identify nursing
activities in pediatric wards information for determining
workload. Rev Esc Enferm USP.

[15] Mitchell ML, Burmeister E, Chaboyer W, Shields L (2012). Psychometrics of the "family-centred care survey- adult
scale. Int J Person Centered Med.

[16] Balbino FS, Balieiro MMFG, Mandetta MA (2016). Measurement of family-centered care perception and parental
stress in a neonatal unit Rev. Latino-Am. Enfermagem.

[17] Pasquali L (2009). Psychometrics. Rev Esc Enferm USP.

[18] Rosana KSM, Marcos AFJ, Diana PSRP, Allyne FV, Viviane EPS, Elizabeth B (2015). Pasquali's model of content validation in the nursing
researches. Rev Enferm.

[19] Pereira RDM, Alvim NAT (2015). Delphi technique in dialogue with nurses on acupuncture as a
proposed nursing intervention. Esc Anna Nery.

[20] Conselho Nacional de Saúde (BR) (2012). Resolução nº 466, de 12 de dezembro de 2012. Regulamenta as pesquisas
com seres humanos.

[21] Matziou V, Chrysostomou A, Vlahiot E, Perdikaris P (2013). Parental presence and distraction during paintful childhood
procedures. Br J Nur.

[22] Buboltz FL, Silveira A, Neves ET, Silva JH, Carvalho JS, Zamberlan KC (2016). Family perception about their presence or not in a pediatric
emergency situation. Texto Contexto- Enferm.

[23] Porter JE, Simon JC, Sellick K (2014). Family presence during resuscitation (FPDR) perceived benefits,
barriers and enablers to implementation and practice. Int Emerg Nurs.

[24] Dall'Orso MS, Concha PJ (2012). Presencia familiar durante la reanimación cardiopulmonar la
mirada de enfermeros y familiares. Cienc Enferm.

[25] Porter J, Dip G, Cooper SJ, Sellick K (2013). Attitudes, implementation and practice of family presence during
resuscitation (FPDR) a quantitative literature review. Int Emerg Nurs.

[26] Goldberger ZD, Nallamothu BK, Nichol G, Chan PS, Curtis JR, Cooke CR (2015). Policies allowing family presence during resuscitation and
patterns of care during in-hospital cardiac arrest. Circ Cardiovasc Qual Outcomes.

[27] Jabre P, Belpomme V, Azoulay E, Jacob L, Bertrand L, Lapostolle F (2013). Family presence during cardiopulmonary
resuscitation. N Engl J Med.

[28] Leske JS, McAndrew NS, Brasel KJ (2013). Experiences of families when present during resuscitation in the
emergency department after trauma. J Trauma Nurs.

[29] Lederman Z, Garasic M, Piperberg M (2014). Family presence during cardiopulmonary resuscitation who should
decide?. J Med Ethics.

[30] Downar J, Kritek PA (2013). Family presence during cardiac resuscitation. N Engl J Med.

[31] Guzzetta C (2016). Family presence during resuscitation and invasive
procedures. Crit Care Nurs.

[32] Tripon Defossez G, Ragot S, Ghazali A, Boureau-Voultoury A, Scépi M, Oriot D (2014). Parental presence during cardiopulmonary resuscitation of
children the experience, opinions and moral positions of emergency teams in
France. Arch Dis Child.

[33] Belpomme V, Adnet F, Mazariegos I, Beardmore M, Duchateau F, Mantz J (2013). Family witnessed resuscitation nationwide survey of 337
prehospital emergency teams in France. Emerg Med J.

[34] Tudor K, Berger J, Polivka BJ, Chlebowy R, Thomas B (2014). Nurses' perceptions of family presence during
resuscitation. Am J Crit Care.

